# Effect of cold arid high-altitude environment on bioactive phytochemical compounds of organically grown Brassicaceae vegetables for nutri-health security in mountainous regions

**DOI:** 10.1038/s41598-024-64926-4

**Published:** 2024-07-10

**Authors:** Shardulya Shukla, Nitish Kumar, Pushpender Bhardwaj, Priyanka Pandita, Manoj Kumar Patel, Mohan Singh Thakur, Raj Kumar, Monisha Rawat, Shweta Saxena

**Affiliations:** 1 Defence Research and Development Organization, Defence Institute of High Altitude Research (DIHAR), C/o 56 APO, Leh-Ladakh, 194101 India; 2 Department of Horticulture, School of Agriculture, Lovely Professional University, Phagwara, Punjab 144411 India; 3 CSIR-IMTECH, GNRPC Building, Sector 39A, Chandigarh, 160036 India; 4 Department of Applied Chemistry, Defence Institute of Advanced Technology (DIAT), Deemed University, Ministry of Defence, Girinagar, Pune, 411025 India

**Keywords:** Organic farming, Brassicaceae, Phytochemical compounds, Antioxidant, Secondary metabolites, Biochemistry, Biological techniques, Ecology, Plant sciences

## Abstract

High-altitude (HA) environment presents immense physiological adversities for humans that have been overcome by supplementing bio-active phytochemicals from functional foods that support and accelerate acclimatization under these extreme environmental conditions. Several agricultural interventions have been investigated to enhance the phytochemical content in vegetables however; these studies have been limited to low-altitude (LA) regions only. In view of an existing knowledge gap, current work is designed to compare the phytochemical compositions of HA and LA-grown Brassicaceae vegetables (cabbage, cauliflower, knol-khol, and radish) using organic treatments via farm yard manure (FYM) and *Azotobacter*. The open field study was conducted as a two-factorial randomized block design. The first factor was treatment (T_1_-FYM, T_2_-*Azotobacter*, T_3_-FYM + *Azotobacter*, and T_4_-control) while the second was locations (HA and LA). Among all these treatments, the application of treatment T_3_ in HA-grown cabbage showed the highest total phenolic content (TPC; 9.56 μg/mg), total flavonoids content (TFC; 14.48 μg/mg), and antioxidant potential using 2,2-diphenyl-1-picrylhydrazyl (DPPH; 85.97%) and ferric reducing antioxidant power (FRAP; 30.77 μg/mg) compared to LA grown samples. Reverse Phase high performance liquid chromatography (RP-HPLC) analysis showed that treatment T_3_ at HA led to significantly high kaempferol (0.92 μg/mg) and sulforaphane (8.94 μg/mg) contents in cabbage whereas, indole-3-carbinol (1.31 μg/mg) was higher in HA grown cauliflower. The present study provides scientific evidence for the enrichment of health-promoting phytochemical compounds in Brassicaceae vegetables grown with T_3_ treatment specifically at HA.

## Introduction

The exposure to high altitude regions such as that of the union territory of Ladakh in India, is well-known for acclimatization adversities faced by sojourners due to multi-factorial physiological challenges^1,2^. The most immediate and damaging impact of the hypobaric hypoxic environment of high altitude is oxidative stress due to increased levels of reactive oxygen species (ROS)^3^. Although an inherent anti-oxidant system combats the oxidative damage sometimes it may not suffice to dampen the damage caused by the overwhelming oxidative stress, thus resulting in the development of high-altitude illnesses of varying degrees such as acute mountain sickness (AMS), high altitude cerebral edema (HACE), and high altitude pulmonary edema (HAPE), etc.^1,3^. Under such situations, supplementation of potent anti-oxidant compounds supports the body’s defense system against the damages caused by ROS. However, serious ramifications along with limited bio-absorption of synthetic anti-oxidants has led to the recently increased exploration of natural and food based anti-oxidant sources^4,5^.

Brassicaceae is a diverse plant family covering about 3500 species and categorized among the most widely consumed vegetables globally encompassing bokchoy, broccoli, brussels sprouts, cabbage, cauliflower, and many more^6,7^. The Brassicaceae plants are naturally rich in bioactive compounds with numerous health benefits including anti-oxidant efficacy^7^. The importance of such health-promoting compounds increases manifolds under adverse climatic conditions such as that of HA regions, making it all the more important to consume bioactive phytochemical compounds rich foods under such situations^8^. Unfortunately, due to the adverse climatic conditions and shorter cultivation periods at HA, most food supplies are met via imports from far-flung low-altitudinal regions, leading to a loss of nutritional quality during long-distance transport^8^. At the same time, excessive usage of chemical fertilizers for enhancing yield and nutritional quality of food crops at HA puts highly vulnerable mountain ecosystems under threat and also affects soil and human health adversely^9^. Thus, there is an urgent need to investigate eco-friendly agricultural interventions to grow nutritionally rich food crops in HA regions to ensure nutri-health security under extreme environmental conditions of HA.

Organic farming has become increasingly popular in the past few decades as it ensures food safety and soil health^10^. Organic manure such as FYM and biofertilizer (*Azotobacter*) not only decreases the need for chemical fertilizers but also provides all the required nutrients to the plants^11,12^. The rhizosphere of plants is covered by a variety of microorganisms, including bacteria and cyanobacteria, which, when applied to the seeds, plant surface, and soil, aids in the conversion of essential nutrients like nitrogen, potassium, and phosphorus from non-absorbable to absorbable forms, which is necessary for the plant’s growth^12,13^. While substantial research has examined the impact of organic farming on the enrichment of phytochemical composition of low-altitude (LA) grown Brassicaceae vegetables, very limited attention has been given to the HA environment where these phytochemicals may play a preventive and therapeutic role against physiological disturbances under extreme environmental conditions. Hence, the present study delves into a comparative analysis of the impact of organic practices on phytochemical composition and anti-oxidant efficacy of Brassicaceae vegetables cultivated in HA vs. LA regions.

## Materials and methods

### Plant sample

Two consecutive year (2020‒2022) field trials were conducted in the open-experimental fields at HA location (Agriculture Research Unit, Defence Institute of High Altitude Research (Leh), India, 3340 mean sea level (msl), 34° 08′ 2″ N; 77° 34′ 3″ E) and LA location (Defence Institute of High Altitude Research, base lab Chandigarh, India, 321 msl, 30° 41′ 31″ N and 76° 47′ 10″ E). Studies were carried out using cruciferous vegetable *i.e*., cabbage (*Brassica oleracea* L. var. *capitata*) cultivar Videshi, cauliflower (*Brassica oleracea* L. var. *botrytis*) cultivar WS909, khol-khol (*Brassica oleracea* L. var. *gongylodes*) cultivar White Vienna and radish (*Raphanus sativus* L.) cultivar Pusa Himani at both HA and LA field locations. Crop seeds were procured from Beejsheetal Research Pvt. Ltd., Mantha Road, Jalna, Maharashtra. The field trials had 12 plots of each vegetable by following a two-factorial randomized block design (2FRBD) with four treatments [(T_1_- FYM @ 150 quintals per hectare (q/ha); T_2_- *Azotobacter* @ 8.6 kg/ha; T_3_- FYM @ 150 q/ha + *Azotobacter* @ 8.6 kg/ha; T_4_- control (without fertilizer)] replicated thrice. For the experiments, a recommended dose of FYM and *Azotobacter* (procured from International Panaacea Ltd.) was used *i.e*. 150 q/ha and 8.6 kg/ha respectively^14^. The area of each plot was 1.62 m^2^ (1.35 m length × 1.20 m width) and a distance of 0.5 m was maintained between adjacent blocks as well as experimental plots. The transplantation of seedlings was done at 2–3 true leaf stage or 15–18 cm height. Plant spacing was maintained for cabbage and cauliflower (60 cm × 45 cm), knol-khol (30 cm × 20 cm), and radish (30 cm × 10 cm) amongst plant-to-plant and line-to-line in all the experimental plots. FYM and *Azotobacter* were applied in each plot before transplanting the seedlings. The field was irrigated by flooding at an interval of 3 days at HA and 6–7 days interval at LA during an early stage of plant establishment, followed by one-week interval (HA) and 2 weeks interval (LA) at later stages. At both locations, there was no use of synthetic fertilizers, pesticides, or herbicides. Weeds were removed manually two to three times during the growing period. The edible portion of cruciferous vegetables was randomly harvested at the maturity stage from each plot. Five kilograms of fresh samples were taken from each treatment and location, shade-dried, well-mixed, and grinded into powder. The powder was then stored at 4 °C in airtight ziplock bags for until further analysis.

### Chemicals

HPLC grade methanol, acetonitrile, acetone, sodium nitrite, sodium hydroxide, and gallic acid were procured from Merck (India). DPPH (1,1-diphenyl-2-picrilhydrazyl), potassium persulfate (PPS), Folin–Ciocalteu (FC) reagent, aluminum chloride, Trolox, quercitin, kaempferol, indole-3-carbinol, sulforaphane, and anion multi-element standards were purchased from the Sigma Aldrich Pvt. Ltd (Switzerland). Sodium bicarbonate, sodium chloride, boric acid, rutintrihydrate, and sodium carbonate were purchased from Himedia (India). The deionized water from the water purification instrument [Merck Millipore Academic, United States of America (USA)] was used for various analyses. All other chemicals were of analytical grade and purchased from Rankem, LobaChemie, and Qualigens Fisher Scientific.

### Sample extraction

The extraction method, duration, temperature, solvent type, and moisture content all play an important role in isolating the essential chemical compounds from plant materials. As a result, a standardized extraction procedure is required for effective yield of desired phytocompounds^15^. In the current research study, 30 g of pulverized sample was extracted thrice via maceration for 24 h at room temperature under dark conditions using 100 ml (each time) of solvent (80% methanol and 20% distilled water). The extracts were filtered to Whatman filter paper grade 1. Further, rotavapor (Buchi R-215, Switzerland) was used to concentrate the filtered extract at a temperature of 40 °C, followed by lyophilization (Esquire biotech Freeze dryer 18N, India) at − 80 °C and 0.050 mbar pressures. These lyophilized extracts were stored in an air-tight container at − 20 °C for further analyses.

### Evaluation of total phenolic content

The total phenolic content (TPC) of sample extracts was determined using the Folin-Ciocalteu (FC) reagent with minor modifications^8^. 70 μL of standard solution (Gallic acid; 2.000–0.332 μg/mL)/extracts (10 mg/mL) were combined in 630 μL of deionized water, followed by the addition of FC reagent (70 μL) and incubation at room temperature for 5 min. In addition, 140 μL of Na_2_CO_3_ solution (20%) was put into each reaction mixture and incubated in the dark conditions for 60 min at room temperature. Following incubation, the absorbance of the samples and standard was measured spectrophotometrically at 750 nm. The results were expressed in μg of Gallic acid equivalent (GAE)/mg of dry powder extract (DPE).

### Evaluation of total flavonoids content (TFC)

TFC was evaluated by the aluminum chloride method with minor modifications^15,16^. 170 μL of standard solution (Rutintrihydrate; 1.46–3.00 μg/mL)/extracts (10 mg/mL) were mixed with 680 μL of deionized water, along with 51 μL of NaNO_2_ (0.72 M) and incubated for 5 min. Subsequently, in each reaction mixture, 51 μL of AlCl_3_ (0.75 M) was added and then incubated for 6 min. Further, 340 μL of NaOH (1.00 M) was added to each reaction mixture. The total reaction volume was made up to 1700 μL by the addition of 408 μL deionized water. Finally, the absorbance was recorded spectrophotometrically at 510 nm. The outcomes were presented in μg of rutin trihydrate equivalent (RE)/mg of DPE.

### Antioxidant activity

#### Evaluation of ferric reducing antioxidant power (FRAP)

The FRAP assay was accomplished as per the technique suggested by Bhardwaj et al.^17^ and Kumar et al.^8^ with minor amendments. Acetate buffer (pH 3.6) 300 mM, TPTZ solution (20 mM in 40 mM HCl), and 20 mM FeCl_3_ (dissolved in water) were mixed in the ratio of 10:1:1 to make FRAP solution, and this FRAP solution was reacted with methanol extract of samples/standard (10.000 mg/mL) in the ratio of 1:30 followed by incubation in the dark conditions (30 min at 37 °C). The blue-colored product (Ferrous tripyridyltriazine complex) was obtained and absorbance was recorded at 593 nm spectrophotometrically. Trolox (0.976—250.000 μg/mL) was used as an assay standard, and outcomes were indicated in μg of Trolox equivalent (TE)/mg of DPE.

#### Evaluation of antioxidant capacity (DPPH radical scavenging activity)

The DPPH radical scavenging activity of extracts was estimated by Zeljkovıc et al.^18^ and Bhardwaj et al.^17^ with minor modifications. DPPH reagent (0.135 mM) was prepared in methanol. The methanolic extracts of test samples (30 mg/mL) / standard (0.480—1.500 μg/mL) were mixed at a ratio of 1:15 with DPPH using a vortex mixer and left at room temperature for 30 min. After incubation, absorbance was measured at 517 nm using a spectrophotometer. Quercetin (QR) was used as a standard. The potential to scavenge radicals was determined by the given formula:

$${\text{Radical scavenging activity }}(\% ) = \frac{{{\text{R}}_{{{\text{sam}}}} - {\text{R}}_{{{\text{sas}}}} }}{{{\text{R}}_{{{\text{sam}}}} }} \times 100$$

R_sam_ = DPPH radical absorbance in methanol; R_sas_ = DPPH radical absorbance in sample/standard.

### Reverse phase high-performance liquid chromatography (RP‑HPLC) analysis

The determination of key phytochemical compounds viz. kaempferol, indole-3-carbinol and sulforaphane was done using RP-HPLC technique (Agilent, Infinity 1200 Series) with photodiode array detector (DAD) as explained by Ahmed et al.^19^ and Kumar et al.^8^ for Kaempferol, Li et al.^20^ for Indole-3-carbinol and Liang et al.^21^ for sulforaphane with some modifications, respectively. Sample peaks, using a sample injection volume of 10 μL, were separated on a Phenomenex C18 column (5 μm, 100 A, 250 × 4.6 mm) maintained at 25 °C temperature with a flow rate of 0.6 mL/min. Before being employed for analysis, all the HPLC quality grade solvents were filtered using a 0.45 µm filter. For kaempferol determination, an isocratic solvent system was deputed using 50% formic acid (0.1%, v/v) and 50% acetonitrile for 18 min with absorbance at 254 nm. For indole-3-carbinol estimation, a gradient elution system was employed by using acetonitrile as mobile phase A and water-formic acid (99.9:0.1, v/v) as mobile phase B with absorbance at 280 nm. The details of the gradient method used were as follows: from 0 to 4 min, 30% mobile phase A; from 4 to 10 min, 50% mobile phase A; from 10 to 12 min, 30% mobile phase A; from 12 to 16 min, 30% mobile phase A. For the determination of sulforaphane, the following mobile phase gradient was used: mobile phase A: acetonitrile; mobile phase B: water-formic acid (99.9: 0.1, v/v) with absorbance at 254 nm. The gradient method used was as follows: from 0 to 4 min, 40% A; from 4 to 10 min, 70% A; from 10 to 12 min, 70% A; from 12 to 20 min, 40% A. Kaempferol, indole-3-carbinol, and sulforaphane standards were used for identification and quantification by making a comparison between RT (retention times) of unspecified peaks with specified standard, and outcomes were presented as μg/mg of DPE.

### Statistical analysis

All analytical assays were repeated thrice and results were compiled as mean ± standard deviation (SD). The data across both consecutive years of the study were pooled (combined) to calculate the average. For determining the significance of the data, viz. results of various phytochemical parameters of Brassicaceae vegetable sample collected from HA and LA experimental fields, an independent t-test and two-way ANOVA were employed at a significance level of *** *p* ≤ 0.001; ** p ≤ 0.01 and * *p* ≤ 0.05 and one-way ANOVA analysis with Duncan’s multiple range tests (*p* < 0.05) was employed in SPSS 16.0 (SPSS Corporation, Chicago, IL)^8^.

### Ethical approval

There is no need of any ethics approval as this investigation was not related with any animal or human subject.

### Plant guideline statement

Experimental research and field studies on plants cultivated, including the collection of plant material, complies with relevant institutional, national, and international guidelines and legislation.

### Consent for publication

All authors have approved the manuscript and agree with its submission to Journal of *Scientific Reports.*

## Result and discussion

### Total phenolic content

Foods derived from plants are rich in polyphenolic compounds, which are effective antioxidants with a plethora of established health benefits, such as anti-inflammatory, anti-mutagenic, and free radical scavenging properties, etc.^8,22^. In the present study, as has been previously reported by Heimler et al.^23^, presence of significant quantities of polyphenolic compounds was demonstrated in all the tested cruciferous vegetables samples grown under different conditions (Table 1). A noteworthy observation of the current investigation was the impact of different organic treatments (FYM and *Azotobacter* alone or in combination) and distinct altitudinal conditions (HA *vs* LA) on the phenolic content of cruciferous vegetables, namely cabbage, cauliflower, knol-khol, and radish. TPC varied from 4.18 to 9.56 µg of GAE mg/DPE. One-way ANOVA analysis indicated that treatment T_3_ showed the highest response in all the different types of test vegetables (cabbage, cauliflower, knol-khol, and radish,). Similar trends were followed by T_2_, T_1_, and T_4_, respectively. Notably, cabbage exhibited significantly higher TPC content in T_3_ treatment at both locations. Furthermore, an independent t-test analysis for TPC content between the HA and LA locations demonstrated a significantly higher content in the HA compared to the LA region. Furthermore, a significant effect of interaction between altitude and treatments (ALT × TRE) was found in the TPC values of cabbage, knol-khol, and radish. The findings of the current study revealed that the T_3_ treatment could maximally boost the TPC values of Brassicaceae vegetables grown at both locations. The higher content of TPC in the T_3_ treatment is most likely due to the cooperative effect of organic manure and plant growth stimulating rhizobacteria (*Azotobacter*) in the biosynthesis that activates the acetate shikimate pathway, resulting in greater phenolics production. These findings are consistent with previous findings of higher TPC levels in organically grown cabbage^24^, broccoli^25^, and cauliflower^26^. Similarly, in another study carried out by Dutta et al.^27^, the phenolic content in turmeric rhizomes was found to be increased when inoculated with rhizobacteria.

**Table 1:** Comparative effect of location and treatments on total phenolic content (µg GAE /mg of DPE) of Brassicaceae vegetables grown at HA versus LA.

ALT	TRE	Cabbage	Cauliflower	Knol-khol	Radish
HA	T_1_	7.61 ± 0.08^bC^***	7.19 ± 0.04^bB^**	6.87 ± 0.14^bA^***	8.05 ± 0.09^bD^***
	T_2_	8.32 ± 0.19^cC^	7.44 ± 0.11^bB^**	7.03 ± 0.23^bA^***	8.34 ± 0.09^cC^***
	T_3_	9.56 ± 0.15^dC^**	8.68 ± 0.20^cB^**	7.97 ± 0.27^cA^***	8.96 ± 0.16^ dB^***
	T_4_	6.27 ± 0.15^aC^**	6.06 ± 0.15^aB^*	5.48 ± 0.05^aA^***	6.62 ± 0.01^aD^***
LA	T_1_	6.88 ± 0.13^bD^	6.69 ± 0.11^bC^	5.11 ± 0.04^bA^	5.71 ± 0.03^bB^
	T_2_	8.27 ± 0.07^cD^	6.96 ± 0.04^cC^	5.42 ± 0.08^cA^	5.81 ± 0.11^bB^
	T_3_	8.91 ± 0.03^dD^	8.07 ± 0.11^dC^	6.55 ± 0.01^dA^	7.18 ± 0.07^cB^
	T_4_	5.73 ± 0.09^aC^	5.73 ± 0.02^aC^	4.18 ± 0.08^aA^	4.63 ± 0.05^aB^
ALT		***	***	***	***
TRE		***	***	***	***
ALT × TRE		***	NS	*	***

HA- high altitude and LA- low altitude, Values presented as means ± SD, ALT: Altitude, TRE: Treatment, T_1_ = FYM @ 150 q/ha, T_2_ = *Azotobacter* @ 8.6 kg/ha, T_3_ = FYM @ 150 q/ha + *Azotobacter* @ 8.6 kg/ha and T_4_ = Control. ALT x TRE—interaction of altitude and treatment. TPC, Total polyphenolic content; GAE, Gallic acid equivalent; DPE, Dry powder extract.

Values in columns different lowercase letters (small alphabet) indicate significantly different; *p* < 0.05, Duncan’s multiple range test between treatments.

Value in row, different uppercase letters (large alphabet) indicate significantly different; *p* < 0.05, Duncan’s multiple range test between the crop.

Mean values in each column (between group) showed significantly different by independent t-test. Two-way ANOVA was applied to visualize the relationship between altitude and treatments. Level of significance: *** *p* ≤ 0.001; ** *p* ≤ 0.01 and * p ≤ 0.05, NS = not significant.

However, it is further noteworthy that despite similar treatments, HA-grown Brassicaceae vegetable samples showed a significantly higher boost in the TPC content than LA-grown vegetables. Plants at higher elevations are exposed to abiotic stresses like overwhelmingly intense UV-B radiation, which has a wide range of effects on plant growth, morphology, and physiology especially triggering different defensive mechanisms which also includes production of polyphenolic secondary metabolites^28,29^. There are few reports such as by Kumar et al.^8^ where it found that extract of *Eruca sativa* samples from high altitude had more phenolic content as compared to low altitude samples. Thus, co-stimulation of plants with abiotic stresses along with organic practices might have lead to the observed rise of polyphenolic secondary metabolite composition. Similarly, Naguib et al.^25^ have also reported that higher abiotic stress in organic farming increased the TPC content in organically grown *Brassica olaracea*, var. *italica*.

### Total flavonoids content

Flavonoids are a sub-category of polyphenols that are highly advised in the nutritionist recommended health promoting diets due to their high efficiency as natural antioxidants as well as preventive and therapeutic properties^30^. The current investigation outlines the impact of different organic treatments on the flavonoid content of Brassicaceae vegetables, namely cabbage, cauliflower, knol-khol, and radish, cultivated at different altitudes. TFC varied from 6.96 to 14.48 µg of rutin trihydrate (RE) per milligram of dry powder extract (DPE) in the current study (Table 2). One-way ANOVA analysis revealed that the treatment T_3_ maximally boosted the flavonoid contents also as it could increase the TPC levels in all the tested Brassicaceae vegetables (cabbage, cauliflower, knol-khol and radish). This trend was also followed by T_2_, T_1_, and T_4_ treatment groups, respectively. Out of these, cabbage exhibited the highest increase in the TFC level in T_3_ treatment at both locations. Overall, cultivation at HA regions supported significantly higher enrichment of TFC, as proved by an independent t-test analysis for TFC content between the HA and LA. A significant interaction between altitude and treatments (ALT × TRE) was found in the TFC of cabbage, cauliflower, and radish (*p* < *0.05*). Similar to the TPC levels, the observed higher content of TFC in the T_3_ treatment can be explained by cooperative effect of FYM and *Azotobacter* treatments in the activation of acetate shikimate biosynthetic pathway^25,27^. These findings are consistent with observations made by earlier researchers where TPC and TFC levels were found to increase with the supplementation of bio-organic fertilizer to cultivated *Brassica oleracea* var. *capitata*^23^, *Brassica oleracea* var. *italica*^25^ and *Brassica oleracea* var. *botrytis*^26^.

**Table 2:** Comparative effect of location and treatments on total flavonoid content (μg RE/mg of DPE) of Brassicaceae vegetables grown at HA versus*,* LA.

ALT	TRE	Cabbage	Cauliflower	Knol-khol	Radish
HA	T_1_	11.95 ± 0.12^bD^***	10.37 ± 0.04^bC^***	9.10 ± 0.07^bB^***	8.68 ± 0.02^bA^***
	T_2_	12.55 ± 0.12^cD^***	10.94 ± 0.02^cC^***	9.42 ± 0.02^cB^***	9.15 ± 0.02^cA^***
	T_3_	14.48 ± 0.41^dD^***	12.34 ± 0.10^dC^***	10.65 ± 0.05^ dB^***	9.88 ± 0.17^dA^**
	T_4_	9.56 ± 0.19^aD^***	9.06 ± 0.03^aC^***	7.99 ± 0.07^aB^***	7.48 ± 0.07^aA^***
LA	T_1_	9.41 ± 0.15^bC^	9.35 ± 0.04^bC^	8.43 ± 0.12^bB^	8.23 ± 0.05^bA^
	T_2_	9.74 ± 0.03^cD^	9.54 ± 0.04^cC^	8.77 ± 0.09^cB^	8.40 ± 0.04^cA^
	T_3_	10.85 ± 0.03^dD^	10.52 ± 0.03^dC^	9.86 ± 0.13^ dB^	9.14 ± 0.05^dA^
	T_4_	7.98 ± 0.16^aD^	8.45 ± 0.02^aC^	7.32 ± 0.05^aB^	6.96 ± 0.09^aA^
ALT		***	***	***	***
TRE		***	***	***	***
ALT × TRE		***	***	NS	**

HA- high altitude and LA- low altitude, Values presented as means ± SD, ALT: Altitude, TRE: Treatment, T_1_ = FYM @ 150 q/ha, T_2_ = *Azotobacter* @ 8.6 kg/ha, T_3_ = FYM @ 150 q/ha + *Azotobacter* @ 8.6 kg/ha and T_4_ = Control. ALT x TRE—interaction of altitude and treatment. DPE, Dry powder extract; TFC, Total flavonoid content; RE, Rutin trihydrate equivalent.

Values in columns different lowercase letters (small alphabet) indicate significantly different; *p* < 0.05, Duncan’s multiple range test between treatments.

Value in row, different uppercase letters (large alphabet) indicate significantly different; *p* < 0.05, Duncan’s multiple range test between the crop.

Mean values in each column (between group) showed significantly different by independent t-test. Two-way ANOVA was applied to visualize the relationship between altitude and treatments. Level of significance: *** *p* ≤ 0.001; ** *p* ≤ 0.01 and * p ≤ 0.05, NS = not significant.

However, as discussed earlier in the manuscript, the key findings of the study demonstrate that HA samples possess significantly higher TFC values than LA samples. Since these secondary metabolites function as part of a plant’s defense mechanisms against abiotic stressors like UV radiations, their raised levels in HA-grown plants are well justified^31^. Our findings are in accordance with the earlier research conducted over a flora of Brassicaceae family (*E. sativa*), Where higher secondary metabolites content was found at HA in comparison to LA^8^ This strategy to boost TFC levels in organically grown vegetables may prove to be a boon to growing anti-oxidant-rich vegetables at HA for local consumption under extreme altitudes that possess a tremendous threat to human health.

### Antioxidant activity

The antioxidant activity of naturally occurring bioactive phytochemicals has been attributed to numerous mechanisms of action, including hydrogen atom transfer, single electron transfer, and their ability to bind transition metals^8,32^. The dietary resource provides an enrichment of a variety of phytochemicals with distinct phenolic groups acting through their unique modes of action in synergistically enhancing the free radical scavengers, crucial in reducing ROS load of the human body^33^. In order to assess the anti-oxidant potentials of LA and HA-grown Brassicaceae vegetables, a combination of two different assays were deployed, *i.e*. DPPH and FRAP, since the full antioxidant potential of a sample cannot be determined by a single experiment due to different mechanisms of actions of different anti-oxidant compounds^34^.

The DPPH assay detects the presence of anti-oxidant compounds which reduce the ROS burden via the mechanism of electron transfer^35^. Thus, DPPH assay was deployed to assess the effect of organic treatments on the free radical scavenging efficacy of various Brassicaceae vegetable samples at different altitudes (Table 3). In the present study, the DPPH scavenging activity varied from 24.74 to 85.97%. As per expectation, higher TPC and TFC levels of T_3_ correspond to the highest DPPH assay based anti-oxidant activity among all the treatments of vegetable samples. The observation of higher anti-oxidant activities in T_3_ plants despite similar growth conditions as T_1_, T_2_, and T_4_ treated plants hints towards a synergistic effect of FYM and *Azotobacter* on secondary metabolites synthesis and their agglomeration. Further, T_3_ treatment of HA demonstrated higher antioxidant activity in comparison to LA which may be due to the higher accumulation of secondary metabolites under abiotic stresses of HA.

**Table 3:** Comparative effect of location and treatments on DPPH content (% inhibition) of Brassicaceae vegetables grown at HA versus LA.

ALT	TRE	Cabbage	Cauliflower	Knol-khol	Radish
HA	T_1_	81.06 ± 0.62^bD^**	79.52 ± 0.34^bC^***	65.99 ± 0.38^bB^***	53.80 ± 0.34^bA^***
	T_2_	82.80 ± 0.22^cD^*	80.95 ± 0.30^cC^***	67.48 ± 0.65^cB^***	55.00 ± 0.20^cA^***
	T_3_	85.97 ± 0.24^dD^***	85.49 ± 0.20^dC^***	71.61 ± 0.26^ dB^***	59.68 ± 0.24^dA^***
	T_4_	65.35 ± 0.25^aC^***	67.18 ± 0.24^aD^***	61.62 ± 0.23^aB^***	32.90 ± 0.22^aA^***
LA	T_1_	78.77 ± 0.58^bD^	64.89 ± 0.27^bC^	60.10 ± 0.25^bB^	34.64 ± 0.06^bA^
	T_2_	80.56 ± 0.85^cD^	65.55 ± 0.80^bC^	61.13 ± 0.23^cB^	35.60 ± 0.51^cA^
	T_3_	82.70 ± 0.50^dD^	70.25 ± 0.60^cC^	64.15 ± 0.47^ dB^	38.71 ± 0.39^dA^
	T_4_	62.23 ± 0.45^aD^	61.26 ± 0.15^aC^	55.82 ± 0.24^aB^	24.74 ± 0.33^aA^
ALT		***	***	***	***
TRE		***	***	***	***
ALT × TRE		NS	***	**	***

HA- high altitude and LA- low altitude, Values presented as means ± SD, ALT: Altitude, TRE: Treatment, T_1_ = FYM @150 q/ha, T_2_ = *Azotobacter* @ 8.6 kg/ha, T_3_ = FYM @ 150 q/ha + *Azotobacter* @ 8.6 kg/ha and T_4_ = Control. ALT x TRE—interaction of altitude and treatment. DPE: Dry powder extract; DPPH: 2, 2-diphenyl-1-picrylhydrazyl assay.

Values in columns different lowercase letters (small alphabet) indicate significantly different; *p* < 0.05, Duncan’s multiple range test between treatments.

Value in row, different uppercase letters (large alphabet) indicate significantly different; *p* < 0.05, Duncan’s multiple range test between the crop.

Mean values in each column (between group) showed significantly different by independent t-test. Two-way ANOVA was applied to visualize the relationship between altitude and treatments. Level of significance: *** *p* ≤ 0.001; ** *p* ≤ 0.01 and * p ≤ 0.05, NS = not significant.

Amongst all these Brassicaceae vegetables, cabbage exhibited a significantly higher DPPH response in T_3_ treatment at HA which also justify the positive correlation of antioxidant activity with TPC and TFC (Table 5). Similar correlation was found in *T. foliolosum* and *E. sativa* between secondary metabolites and their antioxidant activity^8,36^. Plant growth-promoting rhizobacteria (PGPR) are responsible for inducing wide spectrum of systemic resistance via triggering the expression of a battery of genes and pathways to upregulate the accumulation of diverse bioactive molecules^37^. These findings are consistent with observations made by authors where the application of PGPR enhanced the antioxidant capacity of *B. olaracea* L. var. *italica*^24^ and *Glycine max*^38^.

Further, the FRAP test was another anti-oxidant assay deployed to determine specific antioxidants that could reduce Fe^3+^-TPTZ (ferric tripyridyltriazine) into Fe^2+^-TPTZ (ferrous tripyridyltriazine)^17^. The production of the ferrous complex (Fe^2+^-TPTZ) is estimated as the development of the blue-colored complex after reaction incubation^17,39^. Plant extracts with a higher reducing capacity are interpreted as having a higher concentration of antioxidant component^40^. The effect of organic treatments and altitudinal conditions on FRAP assay of various Brassicaceae vegetable samples is shown in Table 4. FRAP assay results were found to vary from 8.61 to 30.77 µg of TE/mg of DPE. On performing a one-way ANOVA analysis, it was found that the T_3_ treatment showed a significantly higher response with respect to all other treatments. Further, an independent t-test analysis for FRAP content between the HA and LA locations demonstrated a significantly higher content in the HA region compared to the LA region. Additionally, cabbage exhibited significantly higher FRAP content in T_3_ treatment at both the locations. A significant interaction between altitude and treatments (ALT × TRE) was found in the FRAP content of cabbage, cauliflower, and radish (*p* < *0.001*). All the above results and correlation analysis (Table 5) indicate that phenolic compounds are as strong contributors for ferric ion chelating activity as they were to DPPH scavenging activity. The study is in strong agreement with the results reported on *E. sativa* and *Onosma riedliana* where a similar relation was found^8,18^.

**Table 4:** Comparative effect of location and treatments on FRAP (μg TE/mg of DPE) content of Brassicaceae vegetables grown at HA versus LA.

ALT	TRE	Cabbage	Cauliflower	Knol-khol	Radish
HA	T_1_	25.41 ± 0.24^bD^***	22.44 ± 0.21^bC^***	17.98 ± 0.32^bB^***	15.19 ± 0.08^bA^***
	T_2_	26.85 ± 0.34^cD^***	24.16 ± 0.16^cC^***	19.02 ± 0.59^cB^*	16.13 ± 0.35^cA^***
	T_3_	30.77 ± 0.46^dD^***	27.34 ± 0.14^dC^***	20.58 ± 0.19^ dB^*	18.12 ± 0.13^dA^***
	T_4_	20.67 ± 0.52^aD^***	19.57 ± 0.06^aC^***	15.03 ± 0.07^aB^***	11.12 ± 0.23^aA^***
LA	T_1_	21.82 ± 0.13^bD^	18.20 ± 0.16^bC^	15.95 ± 0.06^bB^	10.72 ± 0.22^bA^
	T_2_	22.90 ± 0.65^cD^	19.13 ± 0.18^cC^	17.08 ± 0.47^cB^	11.54 ± 0.32^cA^
	T_3_	25.01 ± 0.28^dD^	22.91 ± 0.25^dC^	18.79 ± 0.70^ dB^	13.62 ± 0.25^dA^
	T_4_	16.75 ± 0.19^aD^	16.27 ± 0.17^aC^	13.09 ± 0.38^aB^	8.61 ± 0.12^aA^
ALT		***	***	***	***
TRE		***	***	***	***
ALT × TRE		***	***	NS	***

HA- high altitude and LA- low altitude, Values presented as means ± SD, ALT: Altitude, TRE: Treatment, T_1_ = FYM @ 150 q/ha, T_2_ = *Azotobacter* @ 8.6 kg/ha, T_3_ = FYM @ 150 q/ha + *Azotobacter* @ 8.6 kg/ha and T_4_ = Control. ALT x TRE—interaction of altitude and treatment. DPE: Dry powder extract; TE: Trolox equivalent; FRAP: Ferric reducing antioxidant power assay.

Values in columns different lowercase letters (small alphabet) indicate significantly different; *p* < 0.05, Duncan’s multiple range test between treatments.

Value in row, different uppercase letters (large alphabet) indicate significantly different; *p* < 0.05, Duncan’s multiple range test between the crop.

Mean values in each column (between group) showed significantly different by independent t-test. Two-way ANOVA was applied to visualize the relationship between altitude and treatments. Level of significance: *** *p* ≤ 0.001; ** *p* ≤ 0.01 and * p ≤ 0.05, NS = not significant.

**Table 5:** Correlation between TPC, TFC, FRAP, and DPPH.

	TPC	TFC	FRAP	DPPH
High-altitude				
TPC	1	.617*	0.56	0.275
TFC		1	.971**	.869**
FRAP			1	.943**
DPPH				1
Low-altitude				
TPC	1	.782**	.761**	.661*
TFC		1	.987**	.967**
FRAP			1	.976**
DPPH				1

TPC, Total polyphenolic content; TFC, Total flavonoid content; FRAP, Ferric reducing antioxidant power assay; DPPH, 2, 2-diphenyl-1-picrylhydrazyl assay.

**Correlation is significant at the 0.01 level (2-tailed).

* Correlation is significant at the 0.05 level (2-tailed).

### Effect of different treatments on signature phytochemical compounds

RP-HPLC, which is a reliable and popular chromatographic method for quantifying secondary metabolites in plants^8^, was deployed to develop a comparative profile of secondary metabolites from Brassicaceae plants grown at different altitudes (HA *vs*. LA). The linear regression equations: y = 79691x − 28,706, R^2^ = 0.99, y = 32887x + 65,956, R^2^ = 0.99 and y = 4105x + 27,823, R^2^ = 0.99 were used to calculate the concentration of signature phyto-compounds in Brassicaceae vegetable extracts, for kaempferol (0.122–1000 μg/mL), indole-3-carbinol (0.244–1000 μg/mL) and sulforaphane (7.81–1000 μg/mL) respectively (Table 6,7 & 8).

**Table 6:** Comparative effect of location and treatments on kaempferol content (μg/mg of DPE) of Brassicaceae vegetables grown at HA versus LA.

ALT	TRE	Cabbage	Cauliflower	Radish	Knol-khol
HA	T_1_	0.26 ± 0.01^bA^	0.26 ± 0.00^bA^**	0.47 ± 0.01^bB^***	ND
	T_2_	0.35 ± 0.01^cA^*	0.34 ± 0.01^cA^***	0.46 ± 0.01^bB^***	
	T_3_	0.92 ± 0.02^dC^***	0.81 ± 0.01^ dB^***	0.73 ± 0.01^cA^***	
	T_4_	0.21 ± 0.00^aC^*	0.22 ± 0.00^aB^***	0.29 ± 0.01^aA^***	
LA	T_1_	0.25 ± 0.01^bB^	0.24 ± 0.00^bA^	0.25 ± 0.00^bB^	ND
	T_2_	0.33 ± 0.01^cA^	0.27 ± 0.01^cB^	0.24 ± 0.01^bC^	
	T_3_	0.66 ± 0.01^dA^	0.59 ± 0.02^ dB^	0.32 ± 0.01^cC^	
	T_4_	0.19 ± 0.01^aA^	0.18 ± 0.01^aA^	0.18 ± 0.01^aA^	
ALT		***	***	***	
TRE		***	***	***	
ALT × TRE		***	***	***	

HA- high altitude and LA- low altitude, Values presented as means ± SD, ALT: Altitude, TRE: Treatment, T_1_ = FYM @ 150 q/ha, T_2_ = *Azotobacter* @ 8.6 kg/ha, T_3_ = FYM @ 150 q/ha + *Azotobacter* @ 8.6 kg/ha and T_4_ = Control. ALT x TRE—interaction of altitude and treatment. DPE: Dry powder extract.

Values in columns different lowercase letters (small alphabet) indicate significantly different; *p* < 0.05, Duncan’s multiple range test between treatments.

Value in row, different uppercase letters (large alphabet) indicate significantly different; *p* < 0.05, Duncan’s multiple range test between the crop.

Mean values in each column (between group) showed significantly different by independent t-test. Two-way ANOVA was applied to visualize the relationship between altitude and treatments. Level of significance: *** *p* ≤ 0.001; ** *p* ≤ 0.01 and * p ≤ 0.05, ND = not detect.

**Table 7:** Comparative effect of location and treatments on indole-3-carbinol (μg/mg of DPE) content of Brassicaceae vegetables grown at HA versus LA.

ALT	TRE	Cabbage	Cauliflower	Knol-khol	Radish
HA	T_1_	0.44 ± 0.01^bA^***	1.03 ± 0.02^bC^***	0.42 ± 0.01^bA^***	0.56 ± 0.02^bB^*
	T_2_	0.45 ± 0.02^bA^**	1.08 ± 0.04^cC^***	0.64 ± 0.02^cB^***	0.69 ± 0.04^cB^**
	T_3_	0.65 ± 0.02^cA^***	1.31 ± 0.01^dD^***	0.91 ± 0.02^ dB^***	1.01 ± 0.03^dC^***
	T_4_	0.26 ± 0.01^aB^***	0.22 ± 0.02^aA^**	0.24 ± 0.00^aAB^***	0.36 ± 0.02^aC^***
LA	T_1_	0.31 ± 0.01^bB^	0.22 ± 0.01^bA^	0.30 ± 0.01^bB^	0.50 ± 0.02^bC^
	T_2_	0.34 ± 0.02^cA^	0.34 ± 0.01^cA^	0.50 ± 0.02^cB^	0.61 ± 0.02^cC^
	T_3_	0.52 ± 0.00^ dB^	0.40 ± 0.01^dA^	0.74 ± 0.01^dC^	0.85 ± 0.02^dD^
	T_4_	0.11 ± 0.01^aA^	0.13 ± 0.01^aB^	0.18 ± 0.01^aC^	0.24 ± 0.01^aD^
ALT		***	***	***	***
TRE		***	***	***	***
ALT × TRE		*	***	***	***

HA- high altitude and LA- low altitude, Values presented as means ± SD, ALT: Altitude, TRE: Treatment, T_1_ = FYM @ 150 q/ha, T_2_ = *Azotobacter* @ 8.6 kg/ha, T_3_ = FYM @ 150 q/ha + *Azotobacter* @ 8.6 kg/ha and T_4_ = Control. ALT x TRE—interaction of altitude and treatment. DPE: Dry powder extract.

Values in columns different lowercase letters (small alphabet) indicate significantly different; *p* < 0.05, Duncan’s multiple range test between treatments.

Value in row, different uppercase letters (large alphabet) indicate significantly different; *p* < 0.05, Duncan’s multiple range test between the crop.

Mean values in each column (between group) showed significantly different by independent t-test. Two-way ANOVA was applied to visualize the relationship between altitude and treatments. Level of significance: *** *p* ≤ 0.001; ** *p* ≤ 0.01 and * p ≤ 0.05.

**Table 8:** Comparative effect of location and treatments on sulforaphane (μg/mg of DPE) content of Brassicaceae vegetables grown at HA versus LA.

ALT	TRE	Cabbage	Cauliflower	Knol-khol	Radish
HA	T_1_	2.47 ± 0.05^bB^***	2.74 ± 0.10^bC^**	2.12 ± 0.11^bA^**	2.50 ± 0.10^bB^**
	T_2_	3.06 ± 0.06^cC^***	3.47 ± 0.02^cD^**	1.95 ± 0.06^bA^*	2.46 ± 0.02^bB^***
	T_3_	8.94 ± 0.24^dD^***	4.11 ± 0.02^ dB^***	3.24 ± 0.06^cA^**	4.48 ± 0.04^cC^***
	T_4_	2.04 ± 0.07^aB^***	1.62 ± 0.07^aA^	1.43 ± 0.23^aA^*	1.61 ± 0.03^aA^**
LA	T_1_	2.05 ± 0.04^bB^	2.23 ± 0.12^bC^	1.72 ± 0.03^bA^	1.99 ± 0.07^bB^
	T_2_	1.97 ± 0.09^bB^	2.98 ± 0.13^cC^	1.78 ± 0.06^bA^	1.99 ± 0.09^bB^
	T_3_	4.16 ± 0.05^cD^	3.50 ± 0.04^dC^	2.93 ± 0.05^cA^	3.08 ± 0.11^cB^
	T_4_	1.00 ± 0.07^aA^	1.58 ± 0.07^aC^	0.88 ± 0.08^aA^	1.16 ± 0.10^aB^
ALT		***	***	***	***
TRE		***	***	***	***
ALT × TRE		***	***	*	***

HA- high altitude and LA- low altitude, Values presented as means ± SD, ALT: Altitude, TRE: Treatment, T_1_ = FYM @ 150 q/ha, T_2_ = *Azotobacter* @ 8.6 kg/ha, T_3_ = FYM @ 150 q/ha + *Azotobacter* @ 8.6 kg/ha and T_4_ = Control. ALT x TRE—interaction of altitude and treatment. DPE: Dry powder extract.

Values in columns different lowercase letters (small alphabet) indicate significantly different; *p* < 0.05, Duncan’s multiple range test between treatments.

Value in row, different uppercase letters (large alphabet) indicate significantly different; *p* < 0.05, Duncan’s multiple range test between the crop.

Mean values in each column (between group) showed significantly different by independent t-test. Two-way ANOVA was applied to visualize the relationship between altitude and treatments. Level of significance: *** *p* ≤ 0.001; ** *p* ≤ 0.01 and * p ≤ 0.05.

**Figure 1:**
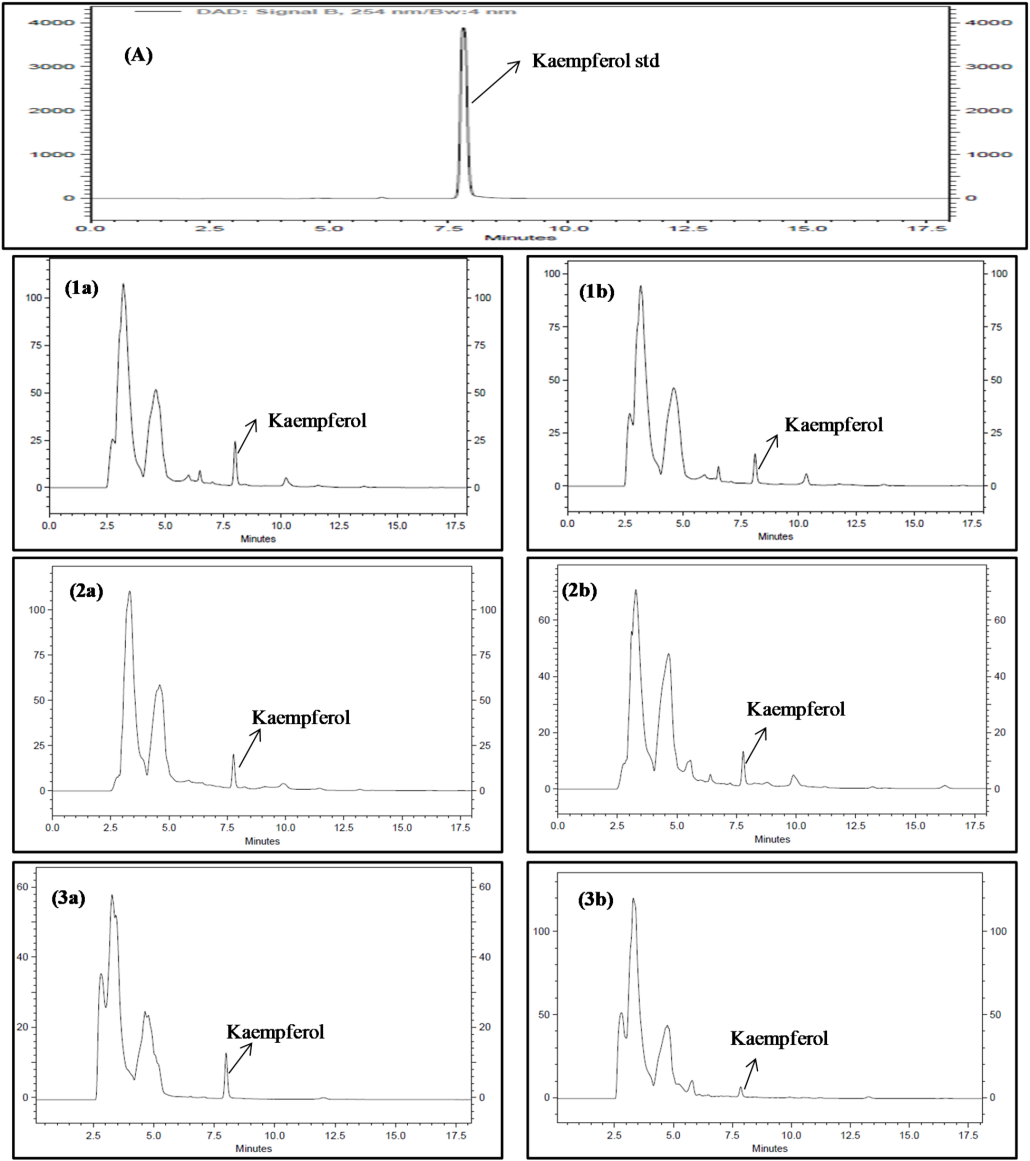
RP-HPLC chromatogram of Brassicaceae vegetables (**A**) Standard peak of kaempferol (**1a**) cabbage: HA_,_ (**1b**) cabbage: LA_,_ (**2a**) cauliflower: HA, (**2b**) cauliflower: LA, (**3a**) radish: HA_,_ (**3b**) radish: LA. HA = High altitude and LA = Low altitude.

Kaempferol is an important signature compound of Brassicaceae family that is known for its anti-cancerous, anti-arthritis, and anti-diabetic properties^19^. The variations in its levels following various organic treatments and also altitudinal conditions were assessed in the present study (Table 6 and Fig. 1). Its levels were found to vary from 0.18 to 0.92 µg/mg of DPE among various test samples. Since kaempferol is a natural flavonol, *i.e.* a type of flavonoid, changes in its levels following different treatments showed trends similar to that of TFC levels. Cabbage exhibited the highest kaempferol content in T_3_ treatment at both locations. A statistically significant correlation was observed between HA and a boost in kaempferol content in Brassicaceae vegetables. Also, altitude and treatments (ALT × TRE) was found to positively interact with kaempferol contents of cabbage, cauliflower, and radish (*p* < *0.001*).

**Figure 2:**
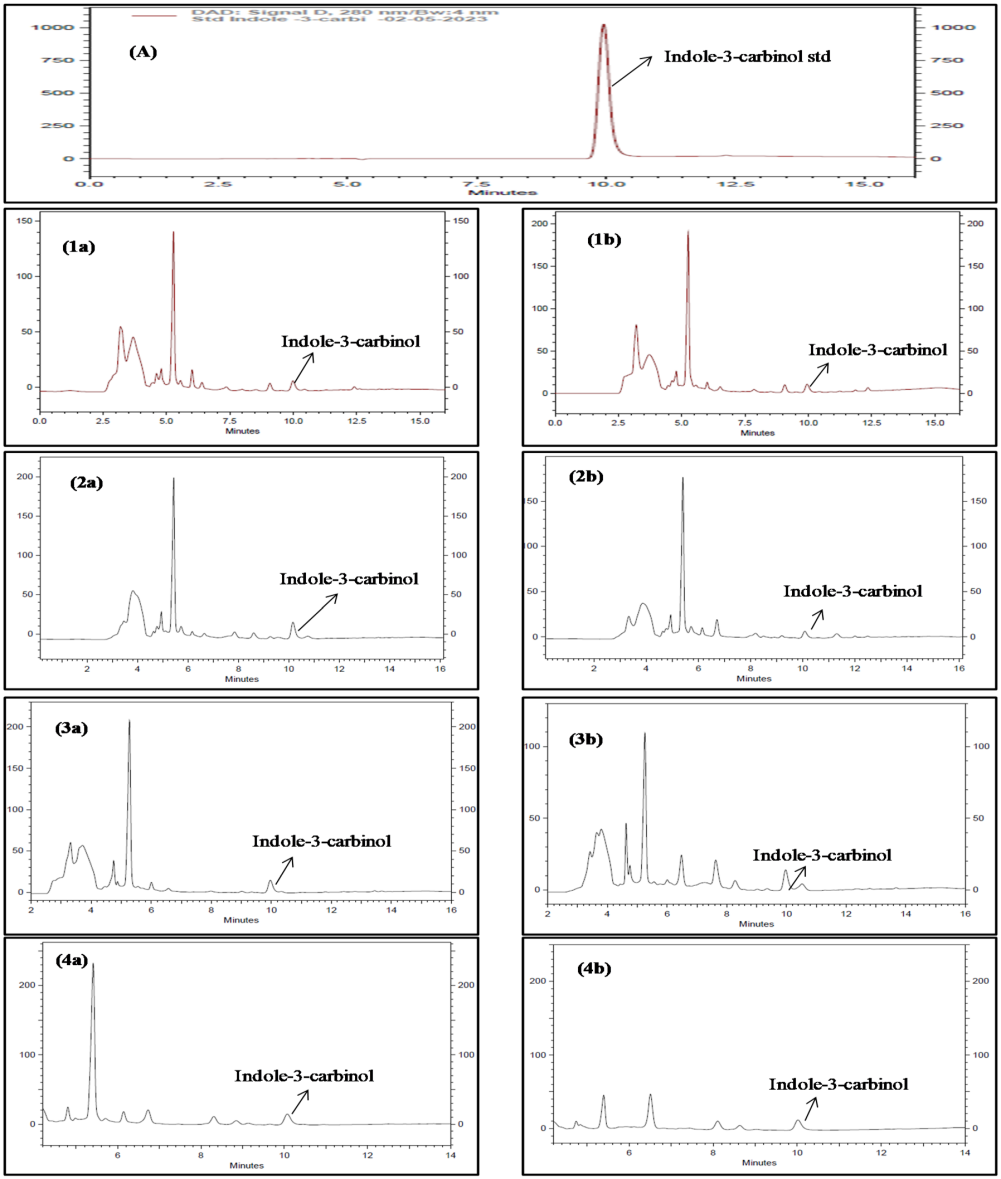
RP-HPLC chromatogram of Brassicaceae vegetables (**A**) Standard peak of indole-3-carbinol (**1a**) cabbage: HA_,_ (**1b**) cabbage: LA_,_ (**2a**) cauliflower: HA, (**2b**) cauliflower: LA, (**3a**) knol-khol: HA_,_ (**3b**) knol-khol: LA, (**4a**) radish: HA_,_ (**4b**) radish: LA. HA = High altitude and LA = Low altitude.

Similarly, the content of another signature compound of Brassicaceae, *i.e.* indole-3-carbinol, was assessed with respect to various organic treatments and altitudinal conditions (Table 7 and Fig. 2). The indole-3-carbinol concentration was found to vary from 0.11 to 1.31 µg/mg of DPE. The treatment T_3_ resulted in maximum accumulation of indole-3-carbinol content in all the Brassicaceae vegetables showing significantly higher contents at HA. Cauliflower showed maximum accumulation of this phytochemical compound in comparison to other tested vegetables. With respect to altitude and interactions with different bio-organic treatments (ALT × TRE) showed similar trends like kaempferol (*p* < *0.05* and *p* < *0.001*).

**Figure 3:**
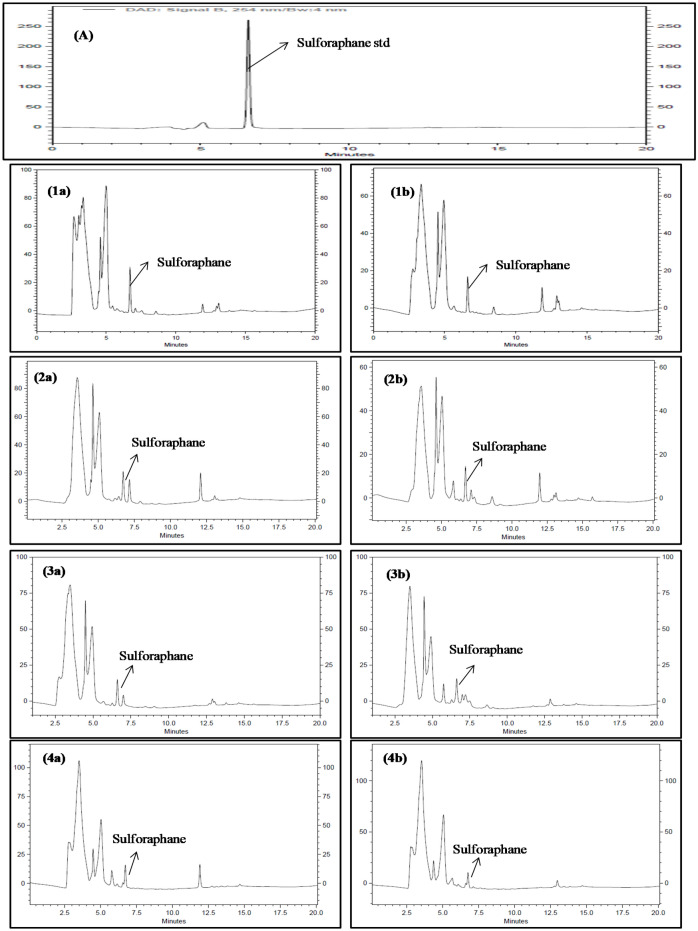
RP-HPLC chromatogram of Brassicaceae vegetables (**A**) Standard peak of sulforaphane, (**1a**) cabbage: HA_,_ (**1b**) cabbage: LA_,_ (**2a**) cauliflower: HA, (**2b**) cauliflower: LA, (**3a**) knol-khol: HA_,_ (**3b**) knol-khol: LA, (**4a**) radish: HA_,_ (**4b**) radish: LA. HA = High altitude and LA = Low altitude.

In addition to this, the vegetable samples were subjected to quantification of another very important signature compound of Brassicaceae vegetables, *i.e*. sulforaphane, which is a sulfur-containing secondary metabolite belonging to isothiocyanates group known for lowering blood pressure, reducing cholesterol levels, and enhancing blood vessel function^21^. Its concentration varied from 0.88 to 8.94 µg/mg of DPE in various test samples (Table 8 and Fig. 3). Among all the studied Brassicaceae vegetables, cabbage showed maximum accumulation of sulforaphane under test conditions. Rest all trends were similar to those obtained for indole-3-carbinol and kaempferol.

Overall, the application of treatment T_3_ (*i.e.* co-treatment of FYM and *Azotobacter*) significantly increased the concentration of all the three tested glucosinolates (*i.e*. kaempferol, indole-3-carbinol and sulforaphane) at both the altitudinal locations. Although these compounds have been earlier reported in Brassicaceae vegetables, the novel finding of our study is that their accumulation is significantly boosted in the HA-grown Brassicaceae vegetables^19,21^. Though the plants synthesize these protective secondary metabolites as part of their defense mechanism under harsh environmental conditions such as extreme temperature, drought, salt, radiation, etc., their dietary enrichment is highly recommended due to their disease-preventing and health-promoting activities in humans. These secondary metabolites are extremely effective in neutralizing reactive oxygen species, thus their regular consumption is linked with reduced incidences of oxidative damage and various inflammatory diseases, including coronary heart disease^41^. At higher elevations, consumption of a diet especially enriched in bioactive phytochemicals is highly recommended to offer protection against highly ionizing environmental conditions. Thus the present study could shed light on effective means to locally produce health-promoting Brassicaceae vegetables at higher elevations using bio-organic techniques.

## Conclusion

The potential of biofertilizers is currently being seriously explored globally as a strategy to reduce the usage of their chemical counterpart and develop an eco-friendly alternative to ensure the nutri-health security of the consumers. The current study has demonstrated that under extreme environmental condition of HA regions, the application of FYM and *Azotobacter* may have a significant impact on the bioactive phytochemical synthesis and accumulation in Brassicaceae vegetables viz*.* cauliflower, cabbage, knol-khol, and radish. The most important finding of the present study is the collaborative effect of FYM and *Azotobacter* (T_3_ treatment) at HA which could lead to the extensive enrichment of bioactive phytocompounds as demonstrated by the HPLC analysis where the quantified glucosinolates (kaempferol, indole-3-carbinol, and sulforaphane) were significantly higher in HA than in LA samples. Similarly, HA-grown Brassicaceae vegetables were found to have higher TPC and TFC values which corroborated with their higher antioxidant potential, in comparison to LA-grown vegetables. A significant correlation was found between TPC, TFC, DPPH, and FRAP assays. Therefore, by means of this study, organic manure combined with biofertilizer is being recommended to grow health promoting Brassicaceae vegetables enriched with specific glucosinolates and other anti-oxidant phytocompounds for local consumption at high altitudes. Further research could be conducted to study the effect of these bio-organic on phytocomponents profile of other families *i.e*., Solanaceae, Cucurbitaceae and Fabaceae at high altitudes.

## Data Availability

All data supporting the findings of this study are available within the paper.

## Abbreviations

DPE: Dry powder extract
TPC: Total phenolic content
TFC: Total flavonoids content
FRAP: Ferric reducing antioxidant power
TPTZ: 2,4,6-Tripyridyl-*s*-triazine
DPPH: 2,2-Diphenyl-1-picrylhydrazyl
Trolox: 6-Hydroxy-2,5,7,8-tetramethylchroman-2-carboxylic acid
GAE: Gallic acid equivalent
RE: Rutin trihydrate equivalent
TE: Trolox equivalent
RT: Retention times
q: Quintal
ANOVA: Analysis of variance
HA: High altitude
LA: Low altitude
MSL: Mean sea level
RP-HPLC: Reverse-phase high performance liquid chromatography
